# The risk and outcome of malignant brain edema in post-mechanical thrombectomy: acute ischemic stroke by anterior circulation occlusion

**DOI:** 10.1186/s40001-023-01414-x

**Published:** 2023-10-13

**Authors:** Luojin Zhang, Jinze Li, Benqiang Yang, Wei Li, Xinrui Wang, Mingyu Zou, Hongyan Song, Lin Shi, Yang Duan

**Affiliations:** 1 Center for Neuroimaging, Department of Radiology, General Hospital of Northern Theater Command, 83 Wenhua Road, Shenhe District, Shenyang, China; 2 Department of Radiology, Shanxi Fenyang Hospital, Shanxi, China; 3 Northern Theater Command Postgraduate Training Base of Jinzhou Medical University General Hospital, Shenyang, China; 4 Department of Radiology, General Hospital of Northern Theater Command, Shenyang, China; 5 Department of Neurology, General Hospital of Northern Theater Command, Shenyang, China

**Keywords:** Acute ischemic stroke, Malignant brain edema, Mechanical thrombectomy, Large vessel occlusion, Hyperdense middle cerebral artery sign

## Abstract

**Background and purpose:**

Malignant brain edema (MBE) occurring after mechanical thrombectomy (MT) in acute ischemic stroke (AIS) could lead to severe disability and mortality. We aimed to investigate the incidence, predictors, and clinical outcomes of MBE in patients with AIS after MT.

**Methods:**

The clinical and imaging data of 155 patients with AIS of anterior circulation after MT were studied. Standard non-contrast CT was used to evaluate baseline imaging characteristics at admission. Clinical outcomes were measured using the 90-day modified Rankin Scale (mRS) score. Based on the follow-up CT scans performed within 72 h after MT, the patients were classified into MBE and non-MBE group. MBE was defined as a midline shift of ≥ 5 mm with signs of local brain swelling. Univariate and multivariate regression analyses were used to analyze the relationship between MBE and clinical outcomes and identify the predictors that correlate with MBE.

**Results:**

MBE was observed in 19.4% of the patients who underwent MT and was associated with a lower rate of favorable 90-day clinical outcomes. Significant differences were observed in both MBE and non-MBE groups: baseline Alberta Stroke Program Early CT (ASPECT) score, hyperdense middle cerebral artery sign (HMCAS), baseline signs of early infarct, angiographic favorable collaterals, number of retrieval attempts, and revascularization rate. Multivariate analysis indicated that low baseline ASPECT score, absent HMCAS, angiographic poor collaterals, more retrieval attempt count, and poor revascularization independently influenced the occurrence of MBE in AIS patients with anterior circulation after MT.

**Conclusion:**

MBE was associated with a lower rate of favorable 90-day clinical outcomes. Low baseline ASPECT score, absent HMCAS, angiographic poor collaterals, more retrieval attempt count and poor revascularization were independently associated with MBE after MT.

## Introduction

Acute ischemic stroke (AIS), caused by large vessel occlusion (LVO), is a common cerebrovascular disease with high morbidity and mortality rates. About 70–80% cases of large vessel occlusion stroke (LVOS) occured in the anterior circulation [1]. Mechanical thrombectomy (MT) was accepted as the standard care for patients with anterior circulation LVOS after findings from five randomized controlled trials (RCTs) were reported in 2015 [2]. Although MT could improve the short-term recanalization rate, patients were still at risk of developing malignant brain edema (MBE), which was a life-threatening complication. Due to the rigidity of the skull, MBE concomitant with intracranial hypertension may not only cause fatal herniation and decrease cerebral perfusion pressure but also compromise cerebral oxygenation and neurological deterioration [3, 4]. Early treatment could help improve the outcomes. Nevertheless, studies on the effect of MT concerning the incidence and predictive factors of MBE were inadequate.

MBE has been associated with several risk factors, such as younger age, higher National Institutes of Health Stroke Scale (NIHSS) score, and larger parenchymal hypoattenuation on CT [4]. Previous studies identified that the hyperdense middle cerebral artery sign (HMCAS) was associated with increased stroke severity at presentation and worse long-term outcomes [5, 6]. However, evidence on the value of HMCAS in predicting the development of MBE and outcomes of MT is limited. Few studies have explored an objective HMCAS measure in patients who undergo MT. Moreover, the interrater reliability of subjective assessment by neurologists remains poor.

Therefore, this study aimed to investigate the incidence, predictors, and clinical outcomes of MBE in AIS patients with anterior circulation large vessel occlusion after MT.

## Materials and methods

### Study population

The Institutional Review Board of the General Hospital of Northern Theatre Command approved the study. All hospitalized patients with anterior circulation LVO ischemic stroke who underwent MT during the period from June 2016 to December 2021 were recruited. Indications for MT followed the most recent treatment guidelines at the time of patient inclusion [7]. Baseline clinical information, imaging data, and periprocedural data of patients were collected and reviewed.

**Fig. 1:**
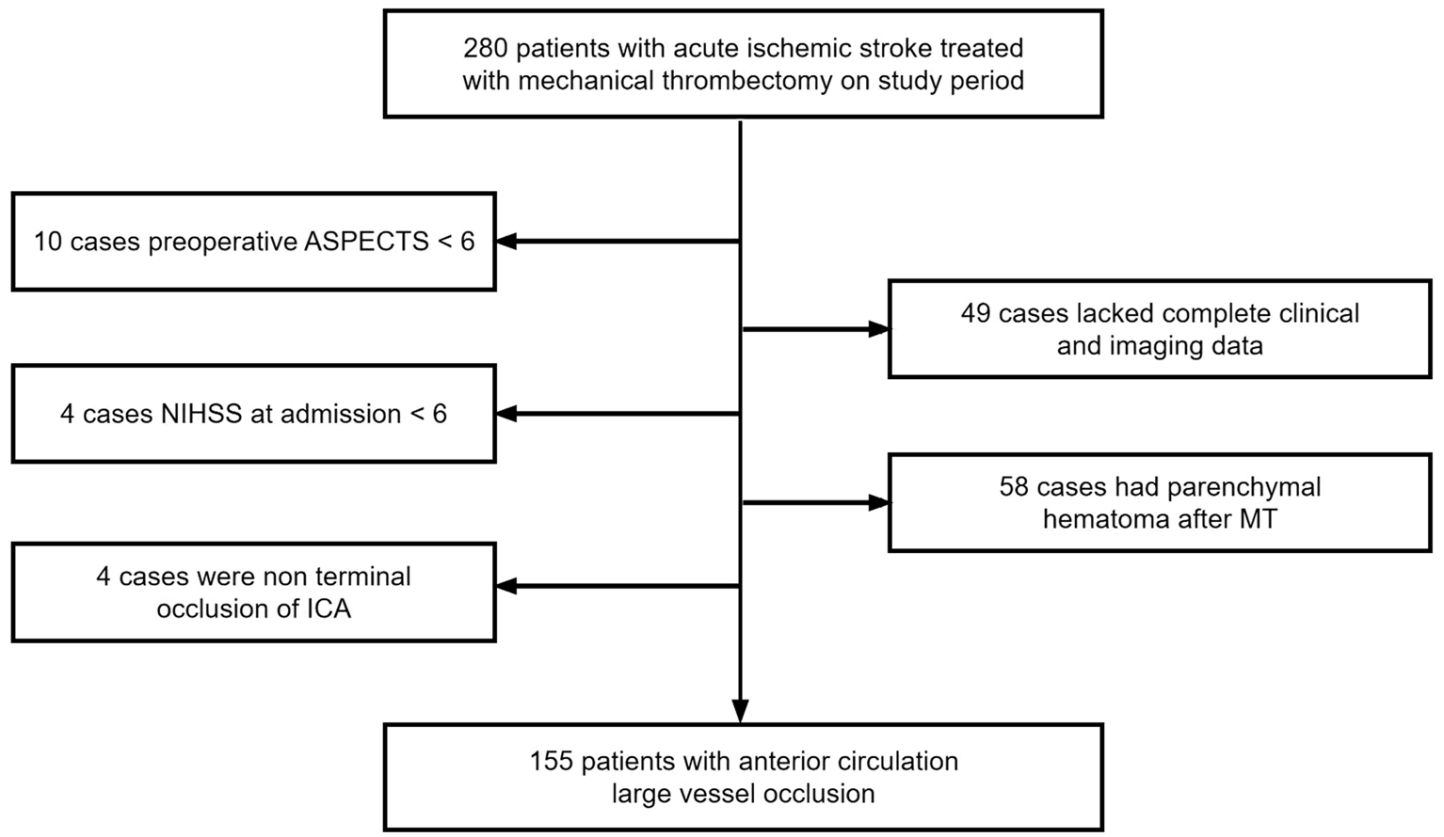
Study flow chart of patients and respective exclusion criteria

The inclusion criteria included: (1) adults aged at least 18 years; (2) anterior circulation LVOS comprising middle cerebral artery (MCA) occlusion (M1 or M2 segments) and terminal internal carotid artery (ICA) occlusion confirmed by digital subtraction angiography (DSA) prior to MT; (3) baseline Alberta Stroke Program Early CT Score (ASPECT) score ≥ 6, baseline NIHSS score ≥ 6, and pre-stroke mRS score ≤ 2; and (4) time from onset to puncture (OTP) ≤ 24 h. The exclusion criteria included: (1) patients with parenchymal hemorrhage (PH) after MT; (2) patients without complete clinical and imaging data. The flowchart of patient enrolment is shown in Fig. 1.

### Clinical information collection and assessment

The baseline characteristics of the study participants were collected from the medical records. They included demographic data (age and sex), medical history (hypertension, diabetes mellitus, coronary heart disease, atrial fibrillation, and past history of stroke), habits and customs (smoking and alcohol use), TOAST, Trial of Org 10172 in Acute Stroke Treatment, radiographic data, and duration from stroke onset to imaging. The periprocedural parameters included occluded vessel region, intravenous thrombolytic therapy, OTP, remedial measures (balloon dilatation and stent implantation), the number of retrieval attempts, and revascularization of occluded vessels. A certified stroke neurologist assessed the severity of the neurological deficit and status of each patient using the NIHSS during admission at the emergency department.

### Image acquisition and analysis

Standard non-contrast computed tomography (NCCT) was performed using a US GE Discovery CT 750 HD scanner (GE Healthcare, Milwaukee, WI, USA). It was initially performed for all participants using the following parameters: 100 kV, 120 mA, 40 mm collimator width, 25 cm field of view, 5 mm layer thickness, and 5 mm layer spacing. Participants were placed in the supine position with their upper limbs lying flat on both sides of their bodies and head slightly inclined.

**Fig. 2:**
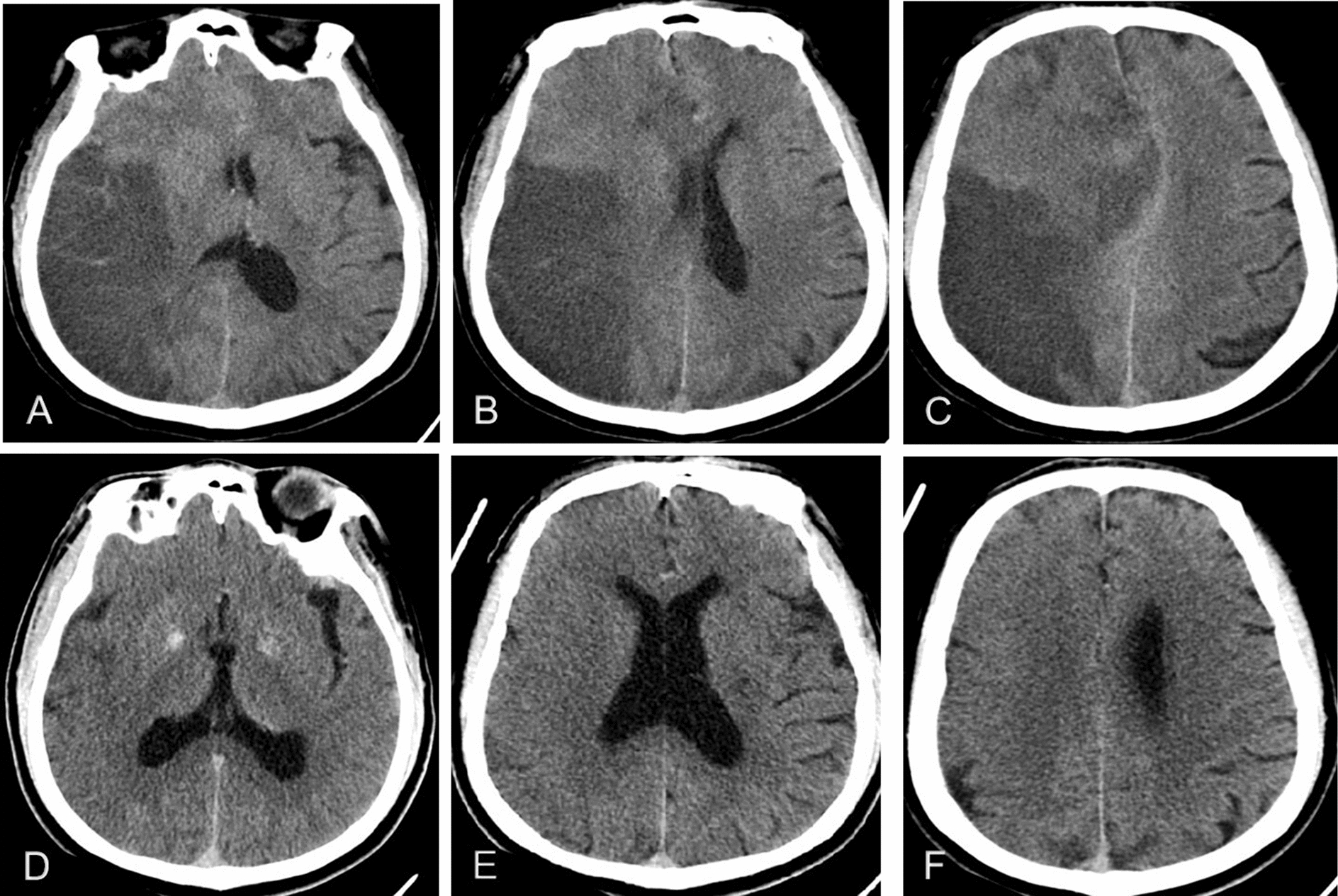
Typical images of MBE after MT: 59-year-old man with mTICI score, 2b; and mRS score, 4; **A**–**C** post-treatment patient with cerebral edema on CT image; **D**–**F** pre-treatment patient without cerebral edema on CT image

Image review was independently performed on a wide-screen high-resolution monitor with an adequate window and level setting. The imaging characteristics of all participants were evaluated by two experienced neuroimaging physicians with no prior knowledge of participants’ clinical information and conventional angiographic findings. In the case of a difference in opinion after assessment, an agreement was reached through consultation. The baseline ASPECT score, HMCAS, and signs of early infarct were assessed on the first NCCT at admission (time from onset to first NCCT ≤ 24 h). MBE was evaluated on NCCT within 72 h after MT. We used the standard ASPECT score to determine stroke burden (range from 0 to 10). A score of zero indicated diffuse cerebral infarction in the region of MCA blood supply, and a score of 10 indicated no new cerebral infarction. To objectively evaluate HMCAS, elliptical regions of interest were placed at MCA on both sides, and measure the attenuation of Hounsfield unit (HU). HMCAS was defined as the MCA attenuation > 43 HU and > 1.2 times of normal contralateral vascular density [8]. Baseline signs of early infarct refer to the observation of any of the following conditions: hypoattenuation in less than one-third of the middle cerebral artery area, blurred lenticular nucleus, disappearance of basal ganglia contour, disappearance of insular ribbon, blurring of sylvian fissure, or disappearance of cortical sulcus [9]. PH was defined as blood clots of the infarcted area with some slight space-occupying effect according to the ECASS (European Collaborative Acute Stroke Study) classification [10]. In previous studies, MBE was present if (1) > 50% of the MCA area had parenchymal hypodensity with signs of local brain swelling, such as disappearance of the sulci and gyri and compression of the lateral ventricle; and (2) midline shift of ≥   5 mm was present at the septum pellucidum or pineal gland with obliteration of the basal cisterns [11] (Fig. 2). The initial angiogram before the treatment allowed for collaterals assessment based on the American Society of Interventional and Therapeutic Neuroradiology/Society of Interventional Radiology [12]. The score ≥ 3 was defined as favorable collateral status.

### Outcome indicators

Intracranial arterial recanalization status was determined by DSA immediately after MT using the modified Thrombolysis in Cerebral Infarction (mTICI) grade. Successful recanalization was defined as an mTICI score of 2b to 3.

Clinical outcomes were measured using mRS at 90 days after onset. A favorable clinical outcome was defined as an mRS of 0–2. An mRS of 2 indicated slight disability (inability to perform all previous activities but cater for themselves without assistance), whereas an mRS ≥ 3 was considered a poor clinical outcome.

### Statistical analysis

The data were analyzed using the SPSS software (version 22.6; IBM, Armonk, New York). Quantitative variables (age, NIHSS score at admission, baseline ASPECT score, time from stroke onset to imaging and OTP, the number of retrieval attempts) were reported as mean ± SD or median (interquartile range [IQR]). Categorical variables (sex, hypertension, diabetes mellitus, coronary heart disease, past history of stroke, atrial fibrillation, smoking, alcohol use, HMCAS, signs of early infarct, TOAST classification, occluded vessel region, intravenous thrombolytic therapy, angiographic favorable collaterals, balloon dilatation, stent implantation, and revascularization) were presented as frequencies and percentages. Normality of distributions was assessed using the Shapiro–Wilk test. The Student’s *t* test was used to compare two groups of normally distributed continuous data, whereas the Mann–Whitney test was used to compare two groups of non-normally distributed continuous data. Fisher’s exact test or *χ*^2^ test was used to compare two groups of categorical data. Univariate and multivariate regression analyses were used to analyze the relationship between MBE and clinical outcomes and identify the predictors of MBE. Factors with *P* ≤ 0.1 in the univariate analysis were included in the logistic regression as independent variables for multivariate analysis. A *P*-value < 0.05 was considered statistically significant.

## Results

### Demography data of patients

Two-hundred and eighty patients with LVO in the anterior circulation and had undergone MT were included in this study. Among these participants, 10 cases had preoperative ASPECT score < 6, 4 cases had NIHSS score at admission < 6, 4 cases had non-terminal occlusion of ICA, 58 cases had parenchymal hematoma, 49 cases lacked complete clinical and imaging data. Finally, 155 patients were included in this study (Fig. 1). The median age of the participants was 64 (IQR, 54–72) years, and 102 (65.8%) participants were male. 79 (51.0%) participants presented with HMCAS, and 81 (52.3%) participants presented with baseline signs of early infarct. Participants’ baseline ASPECT score was 8 (IQR, 8–10), and NIHSS score at admission was 14 (IQR, 11–17). 41 (26.5%) participants received MT and intravenous thrombolysis, and 130 (83.9%) participants were revascularized successfully. 68 (43.9%) participants had favorable 90-day clinical outcomes (mRS ≤ 2) (Table 1).

**Table 1:** Comparison between MBE and non-MBE groups after MT in patients with ALVOS

	All patients (*N* = 155)	non-MBE group (*N* = 125)	MBE group (*N* = 30)	*P*
Age, median (IQR), years	64(54–72)	65(54–73)	61(56.25–69.5)	0.231
Male, *n* (%)	102(65.8)	83(66.4)	19(63.3)	0.750
Medical history, *n* (%)				
Hypertension	94(60.6)	74(59.2)	20(66.7)	0.452
Diabetes mellitus	41(26.5)	36(28.8)	5(16.7)	0.176
Coronary heart disease	29(18.7)	26(20.8)	3(10.0)	0.173
Past history of stroke	36(23.2)	31(24.8)	5(16.7)	0.343
Atrial fibrillation	53(34.2)	46(36.8)	7(23.3)	0.163
Smoking	77(49.7)	62(49.6)	15(50.0)	0.969
Alcohol use	71(45.8)	56(44.8)	15(50.0)	0.608
Clinical and imaging characteristics				
NIHSS score at admission, median (IQR), score	14(11–17)	14(11–17)	16(12–19)	0.074
Baseline ASPECT score, median (IQR), score	8(8–10)	9(8–10)	8(6–9)	< 0.001
Present HMCAS, *n* (%)	79(51.0)	73(58.4)	6(20.0)	< 0.001
Signs of early infarction, *n* (%)	81(52.3)	57(45.6)	24(80.0)	0.001
TOAST classification, *n* (%)				0.572
LAA	97(62.6)	77(61.6)	20(66.7)	
Cardioembolic	42(27.1)	36(28.8)	6(20.0)	
Undetermined or others	16(10.3)	12(9.6)	4(13.3)	
Occluded vessel region, *n* (%)				0.193
ICAT	29(18.7)	20(16.0)	9(30.0)	
MCA M1	85(54.8)	73(58.4)	12(40.0)	
MCA M2	13(8.4)	11(8.8)	2(6.7)	
ICA + MCA	28(18.1)	21(16.8)	7(23.3)	
Time from onset to imaging, median (IQR), min	187(105–336)	203(106.5–349.5)	159.5(87–292.75)	0.305
OTP, median (IQR), min	355(195–524)	375(206–545)	273(176.75–460.25)	0.162
Intravenous thrombolysis, *n* (%)	41(26.5)	33(26.4)	8(26.7)	0.976
Angiographic favorable collaterals, *n* (%)	54(34.8)	49(39.2)	5(16.7)	0.020
Number of retrieval attempts, median (IQR), count	1(1–2)	1(1–2)	2(1–4)	0.018
Balloon dilatation, *n* (%)	18(11.6)	16(12.8)	2(6.7)	0.532
Stent implantation, *n* (%)	7(4.5)	6(4.8)	1(3.3)	1.000
Revascularization (mTICI ≥ 2b), *n* (%)	130(83.9)	115(92.0)	15(50.0)	< 0.001
Favorable clinical outcome (mRS ≤ 2), *n* (%)	68(43.9)	64(51.2)	4(13.3)	< 0.001

MBE, malignant brain edema; NIHSS, National Institutes of Health Stroke Scale; ASPECT, Alberta Stroke Program Early CT; HMCAS, hyperdense middle cerebral artery sign; TOAST, Trial of Org 10172 in Acute Stroke Treatment; LAA, large-artery atherosclerosis; ICAT, internal carotid artery terminal; MCA M1, middle cerebral artery M1 segment; MCA M2, middle cerebral artery M2 segment; ICA, internal carotid artery; MCA, middle cerebral artery; OTP, time from onset to puncture; mTICI, modified Thrombolysis in Cerebral Infarction; mRS, modified Rankin Scale

### Factors associated with MBE

MBE was observed in 30 (19.4%) participants. The MBE group recorded a lower baseline ASPECT score than the non-MBE group (8 [6–9] vs 9 [8–10], *P* < 0.001). The MBE group had a lower HMCAS (20.0% vs 58.4%, *P* < 0.001), angiographic favorable collateral (16.7% vs 39.2%, *P* = 0.020) and revascularization rate (50.0% vs 92.0%, *P* < 0.001) than the non-MBE group. Additionally, the MBE group recorded higher baseline signs of early infarct (80.0% vs 45.6%, *P* = 0.001) and greater number of retrieval attempts (2 [1–4] vs 1 [1, 2], *P* = 0.018) than the non-MBE group.

No between-group differences were observed in age, sex, medical history, baseline NIHSS score, TOAST classification, intravenous thrombolytic therapy, stent implantation, and remedial measures including balloon dilatation. Additionally, no differences were observed in the occluded vessel region, time from onset of stroke to imaging, OTP, and medical history between both groups (*P* > 0.05) (Table 1).

Binary logistic regression analysis showed that low baseline ASPECT score (OR = 0.429, 95% CI 0.238–0.773, *P* = 0.005), absent HMCAS (OR = 4.576, 95% CI 1.352–15.485, *P* = 0.014), angiographic poor collaterals (OR = 5.346, 95% CI 1.322–21.628, *P* = 0.019), more retrieval attempt count (OR = 1.877, 95% CI 1.016–3.465, *P* = 0.044), and poor revascularization (mTICI < 2b) (OR = 11.937, 95% CI  2.932–48.602, *P* = 0.001) were independently associated with MBE after MT in patients with AIS caused by an anterior circulation LVO. Adjustment for the following confounding factors was performed before the analysis: NIHSS score at admission, baseline ASPECT score, absent HMCAS, baseline signs of early infarct, angiographic poor collaterals, poor revascularization, and number of retrieval attempts (Table 2).

**Table 2:** Multivariate analysis of MBE after MT for AIS caused by anterior circulation LVO

	*P*	OR	95% CI
NIHSS score at admission	0.723	1.020	0.916–1.135
Baseline ASPECT score	0.005	0.429	0.238–0.773
Absent HMCAS	0.014	4.576	1.352–15.485
Signs of early infarction	0.669	1.409	0.293–6.767
Angiographic poor collaterals	0.019	5.346	1.322–21.628
Number of retrieval attempts	0.044	1.877	1.016–3.465
Poor revascularization	0.001	11.937	2.932–48.602

NIHSS, National Institutes of Health Stroke Scale; ASPECT, Alberta Stroke Program Early CT; HMCAS, hyperdense middle cerebral artery sign

### MBE and clinical outcomes

In the univariate analysis, patients without MBE had better favorable 90-day clinical outcomes than patients with MBE (51.2% vs 13.3%, *P* < 0.001). Binary logistic regression analysis showed that patients with MBE (OR = 0.284, 95% CI  0.083–0.969, *P* = 0.044) were inversely associated with favorable 90-day clinical outcomes after adjusting for the NIHSS score at admission, angiographic favorable collaterals, number of retrieval attempts, revascularization, and presence of MBE (Tables 3 and 4).

**Table 3:** Comparison between favorable clinical outcome and poor clinical outcome groups after MT

	Favorable clinical outcome (mRS ≤ 2) (*N* = 68)	Poor clinical outcome (mRS ≥ 3) (*N* = 87)	*P*
Age, mean ± SD, years	62.59 ± 11.19	64.52 ± 13.43	0.342
Male, *n* (%)	44(64.7)	58(66.7)	0.798
Medical history, *n* (%)			
Hypertension	42(61.8)	52(59.8)	0.801
Diabetes mellitus	16(23.5)	25(28.7)	0.466
Coronary heart disease	16(23.5)	13(14.9)	0.174
Past history of stroke	14(20.6)	22(25.3)	0.492
Atrial fibrillation	22(32.4)	31(35.6)	0.669
Smoking	35(51.5)	42(48.3)	0.693
Alcohol use	28(41.2)	43(49.4)	0.306
Clinical and imaging characteristics			
NIHSS score at admission, median (IQR), score	13(11–16)	15(12–18)	0.007
Baseline ASPECT score, median (IQR), score	8.5(8–10)	8(7–10)	0.501
Present HMCAS, *n* (%)	39(57.4)	40(46.0)	0.160
Signs of early infarction, *n* (%)	32(47.1)	49(56.3)	0.252
TOAST classification, *n* (%)			0.701
LAA	45(66.2)	52(59.8)	
Cardioembolic	17(25.0)	25(28.7)	
Undetermined or others	6(8.8)	10(11.5)	
Occluded vessel region, *n* (%)			0.473
ICAT	12(17.6)	17(19.5)	
MCA M1	40(58.8)	45(51.7)	
MCA M2	7(10.3)	6(6.9)	
ICA + MCA	9(13.2)	19(21.8)	
Time from onset to imaging, median (IQR), min	196(108.25–341.75)	181(100–336)	0.861
OTP, median (IQR), min	387.5(216.75–502.25)	345(185–560)	0.660
Intravenous thrombolysis, *n* (%)	16(23.5)	25(28.7)	0.466
Angiographic favorable collaterals, *n* (%)	37(54.4)	17(19.5)	< 0.001
Number of retrieval attempts, median (IQR), count	1(1–2)	2(1–2)	0.009
Balloon dilatation, *n* (%)	11(16.2)	7(8.0)	0.117
Stent implantation, *n* (%)	4(5.9)	3(3.4)	0.700
Revascularization (mTICI ≥ 2b), *n* (%)	64(94.1)	66(75.9)	0.002
MBE, *n* (%)	4(5.9)	26(29.9)	< 0.001

mRS, modified Rankin Scale; NIHSS, National Institutes of Health Stroke Scale; ASPECT, Alberta Stroke Program Early CT; HMCAS, hyperdense middle cerebral artery sign; TOAST, Trial of Org 10172 in Acute Stroke Treatment; LAA, large-artery atherosclerosis; ICAT, internal carotid artery terminal; MCA M1, middle cerebral artery M1 segment; MCA M2, middle cerebral artery M2 segment; ICA, internal carotid artery; MCA, middle cerebral artery; OTP, time from onset to puncture; mTICI, modified Thrombolysis in Cerebral Infarction; MBE, malignant brain edema

**Table 4:** Multivariate analysis of favorable clinical outcome after MT for AIS caused by anterior circulation LVO

	P	OR	95% CI
NIHSS score at admission	0.107	0.929	0. 850–1.016
Angiographic favorable collaterals	< 0.001	4.318	1.995–9.346
Number of retrieval attempts	0. 058	0. 610	0. 366–1.016
Revascularization	0. 154	2.646	0. 694–10.096
MBE	0. 044	0. 284	0. 083–0. 969

NIHSS, National Institutes of Health Stroke Scale; MBE, malignant brain edema

## Discussion

This study investigated the incidence, risk factors, and clinical outcomes of MBE in patients with AIS due to anterior circulation LVO and had undergone MT. The incidence of MBE was 19.4% in our study. MBE was associated with poor clinical outcomes (mRS ≥ 3). Participants with absent HMCAS were more likely to develop MBE after MT. Moreover, low baseline ASPECT score, angiographic poor collaterals, more retrieval attempt count and poor revascularization (mTICI < 2b) were significant predictors of malignant progression.

A large multi-center study on a cohort of patients with AIS and LVO of anterior circulation showed that the MBE occurrence rates differed significantly among treatment groups. The treatment groups—no treatment, IV-TPA, and EVT—recorded rates of 50.1%, 32.2%, and 23.8%, respectively [13]. The results showed that intravascular therapy can effectively reduce the incidence of MBE. According to our results, the occurrence rate of MBE in MT-treated patients with AIS due to anterior circulation LVO was 19.4%. MBE was associated with poor prognosis. Therefore, early identification of high-risk patients with MBE after MT is necessary deciding the treatment options, which can guide clinicians to initiate life-saving treatment early to improve the prognosis of patients' neurological function.

A hyperdense segment of vessels, which represented direct imaging of the intravascular thrombus, was detected on NCCT in patients with AIS. Although this segment could be observed in any vessel, it was mostly observed in MCA as HMCAS [14]. HMCAS was found in only 33–50% of angiographically confirmed cases of thrombosis [14]. The HMCAS was observed in 51.0% (79/155) patients in our study, which may be related to the time of the CT scan after onset of cerebral infarction, thickness of CT layer, and composition of thrombosis. The HMCAS on head CT may be observed as early as within 90 min after ictus and disappears within few days [15, 16]. Manelfe et al. [17] found that in 107 patients with present HMCAS within 6 h, 55% of patients showed present HMCAS in the second CT scan within 12–24 h, whereas only 26% of patients showed present HMCAS in the third CT scan within 6–8 days. AIS thrombi consist of three components: red blood cells (RBCs), white blood cells (WBCs), and fibrin/other components. According to previous studies, the presence and density of HMCAS were associated with RBC-dominant thrombi, which represented the concentration of hemoglobin, whereas the absence of HMCAS was indicative of fibrin/platelet-rich thrombi [18].

Our results showed that absent HMCAS was related to the occurrence of MBE (Table 2). We speculate that the patients were more prone to MBE in the absence of HMCAS due to the composition of the embolus. Thrombus components have different characteristics, such as stiffness, stickiness, deformability, and mechanical friction. All these characteristics can influence the success rate of recanalization. RBC-rich thrombi had a lower coefficient of friction, lower stiffness, potentially higher deformability, and better integration ability of stent strut into the thrombus than fibrin/platelet-rich thrombi [18]. The degree of clot integration into the thrombectomy device was decreased in fibrin-rich thrombi. The fibrin strands could increase clot rigidity and influence the thrombus coefficient of friction and level of physical compression, making these thrombi more resistant to mechanical removal.

Previous studies found that cardioembolic thrombi were characterized by higher amounts of RBCs and lower amounts of fibrin than large-artery atherosclerosis (LAA) thrombi [18]. Kim et al. [19] found that aortic atherosclerosis was more common in patients with absent HMCAS. Therefore, we speculate that absent HMCAS on NCCT before MT may indicate in situ thrombotic occlusion caused by large atherosclerosis and poor vascular recanalization rate. We thought that radiological imaging could predictably characterize thrombus composition and determine thrombus location and size. Current imaging modalities could not only differentiate between RBC-dominant and fibrin/platelet-dominant thrombi but also guide clinical decisions, such as selection of thrombus-specific retrieval protocols or device technologies. Future studies will determine the link between radiological signs and thrombus composition and develop pre-treatment decision-making strategies to improve the success rates of first-pass recanalization.

Our results showed that more retrieval attempt count were associated with poor functional outcomes. More retrieval attempt count increases the risk of vascular injury and destruction of the blood–brain barrier due to MBE [20]. Available evidence informing the ability of retrieval attempts number to reliably predict the development of MBE are insufficient. Our results showed that the number of retrieval attempts was a potential biomarker for predicting MBE after MT.

Additionally, we found that low ASPECT score was an independent factor associated with MBE, which was consistent with findings from previous studies [21–23]. The ASPECT score was a scoring system that can identify the extent of the initial infarct on CT after a stroke. Although the detection methods of ischemic size in these studies were different (multiphase perfusion CT, magnetic resonance diffusion weighted imaging, and NCCT), all the results showed that the ischemic size at baseline was related to MBE.

Previous studies have shown that the range of the pial collateral was related to the infarct growth and final infarct volume, and the distribution of undesirable collateral was closely related to the severity of hypoperfusion [24–26], in line with our findings.

In acute LVOS, early restoration of blood flow to ischemic brain tissues had repeatedly been shown to improve clinical outcomes. However, several studies have suggested that revascularization may lead to secondary injury [27, 28]. After cerebral vascular recanalization with intra-arterial thrombectomy, occluded vascular recanalization causes more water to pass through the damaged blood–brain barrier. This aggravates angiogenic edema, further aggravates the destruction of the blood–brain barrier, triggers secondary cascade injury reaction, and leads to neuronal death. Additionally, the results of previous animal experiments showed that recanalization may aggravate the development of brain edema [29–31]. Contrarily, other studies had shown that successful recanalization could reduce the development of early and late brain edema [32–34]. The recovery of cerebral blood flow may save brain tissues by saving ischemic penumbra, preventing the growth of infarct size, and limiting edema-related tissue damage [32]. Therefore, the occurrence degree and injury outcomes of edema might depend on the balance between benefit of vascular recanalization and reperfusion brain edema injury.

Findings from our study indicated that successful recanalization reduced the occurrence of MBE within 72 h. Post-hoc analysis of the MR-CLEAN cohort showed that [34] successful recanalization was associated with a reduced likelihood of midline displacement on a 24-h follow-up CT. Thorén et al. [33] found that successful recanalization of blood vessels reduced the occurrence of moderate-to-severe brain edema within 22–36 h. Huang et al. [11] evaluated the midline shift on the follow-up images within 72 h, and the results showed that the vascular recanalization rate in the MBE group was significantly lower than that in the non-MBE group, which is consistent with findings from our study. Irvine et al. [32] reported that reperfusion was associated with smaller midline shift and lower swelling volume when MRI was taken during 3–8 days of follow-up. On all accounts, although the destruction of the blood–brain barrier may be exacerbated after vascular recanalization, this treatment method may reduce the risk and degree of MBE.

This study had some limitations. First, all patients were recruited from a single institution, limiting the sample size available and generalizability of the outcomes. Second, the definition and evaluation of brain edema were relatively simple (imaging-based NCCT). Third, we excluded patients with PH, consistent with prior studies [11, 34–36], which will lead to some patients with coexisting edema and hemorrhage were also exclude. Finally, due to lack of CTP or DWI examinations in certain patients before MT, cerebrovascular reserve calculation in CT/MR perfusion and DWI core volume were not considered in this study. Further multi-center prospective large population studies, considering more clinical risk factors, are needed to confirm our results.

## Conclusion

The incidence of MBE after MT was 19.4% in this study. MBE was associated with a lower rate of favorable 90-day clinical outcomes in patients with AIS who had an anterior circulation LVO after MT. Absent HMCAS, low baseline ASPECT score, angiographic poor collaterals, more retrieval attempt count and poor revascularization (mTICI < 2b) were independently associated with the occurrence of MBE after MT.

## Data Availability

The datasets analyzed during the current study are not publicly available due to intellectual property rights, but are available from the corresponding author on reasonable request.

## Abbreviations

MBE: Malignant brain edema
MT: Mechanical thrombectomy
AIS: Acute ischemic stroke
mRS: Modified Rankin Scale
ASPECT: Alberta Stroke Program Early CT
LVO: Large vessel occlusion
LVOS: Large vessel occlusion stroke
RCTs: Randomized controlled trials
NIHSS: National Institutes of Health Stroke Scale
HMCAS: Hyperdense middle cerebral artery sign
MCA: Middle cerebral artery
ICA: Internal carotid artery
DSA: Digital subtraction angiography
OTP: Time from onset to puncture
PH: Parenchymal hemorrhage
ECASS: European Collaborative Acute Stroke Study
TOAST: Trial of Org 10172 in Acute Stroke Treatment
NCCT: Non-contrast computed tomography
HU: Hounsfield unit
mTICI: Modified Thrombolysis in Cerebral Infarction
RBCs: Red blood cells
WBCs: White blood cells
LAA: Large-artery atherosclerosis
